# Wearable Technology for “Real-World Research”: Realistic or Not?

**DOI:** 10.1177/0301006620928324

**Published:** 2020-06-18

**Authors:** Roy S. Hessels, Diederick C. Niehorster, Gijs A. Holleman, Jeroen S. Benjamins, Ignace T. C. Hooge

**Affiliations:** Department of Experimental Psychology, Helmholtz Institute, Utrecht University, the Netherlands; Department of Developmental Psychology, Utrecht University, the Netherlands Email:royhessels@gmail.com; Lund University Humanities Lab, Lund University, Sweden; Department of Psychology, Lund University, Sweden; Department of Experimental Psychology, Helmholtz Institute, Utrecht University, the Netherlands; Department of Developmental Psychology, Utrecht University, the Netherlands; Department of Experimental Psychology, Helmholtz Institute, Utrecht University, the Netherlands; Department of Social, Health and Organisational Psychology, Utrecht University, the Netherlands; Department of Experimental Psychology, Helmholtz Institute, Utrecht University, the Netherlands

Research conducted in laboratories is unlikely to be representative of human functioning in the “real world.” Or so one is led to believe when one sifts through the psychological literature of the last century. Discussions about the generalisability of laboratory research beyond its confines have arisen in the fields of perception, attention, memory, and social cognition as well as many applied sciences (see Holleman et al., 2020b, for an overview). More often than not, papers advocating research in the “real world” are published in high-impact journals. Of course, this is in itself no reason for particular concern. What does concern us is that some of these papers make unrealistic claims about how “real-world research”—whatever its precise definition—is to be conducted. The fact that such claims are made in high-impact journals is worrisome, as papers in these journals are often the basis for grant applications or serve as a starting point for new researchers.

A recent claim that particularly concerns us is that techniques such as wearable eye trackers, mobile electroencephalography (EEG), and mobile functional near-infrared spectroscopy (fNIRS) will allow researchers to finally conduct “real-world research” (Pérez-Edgar et al., 2020; Shamay-Tsoory & Mendelsohn, 2019). The gist is that with wearable technology, one can freely roam the world, while (eye-movement) behaviour and its neural correlates are continuously measured, allowing for profound insights into human perception and cognition. In our view, there is a stark contrast between how this claim is presented and the real problems of the empirical work. We have often found that researchers vastly underestimate the reality of conducting research with wearable technology, whether it is conducted inside or outside of the laboratory. Using wearable eye tracking as an example, we show that there are theoretical, conceptual, and methodological problems when moving from classical laboratory-based research to research using wearable technology, which many researchers seem to be unaware of. Although our focus is on eye-tracking research, as that is where our expertise lies, we expect that similar concerns can be raised about research using mobile EEG or mobile fNIRS.

The fact that some researchers overestimate what wearable eye trackers can deliver is perhaps best illustrated by a quote from a recent paper advocating the use of wearable technology for “real-world research.” Shamay-Tsoory & Mendelsohn (2019) state that “… *newly available portable eye-tracking systems offer a cost-effective, easy to apply, and reliable measure of eye gaze and saccades in an ecological* environment” (p. 853, our emphasis). If this were true, wearable eye trackers might indeed be wonderful tools for studying “real-life” behaviour. However, we contest these three postulated characteristics of wearable eye trackers. Moreover, in our view, these beliefs are some of the most common misconceptions about wearable eye trackers.

## Is Wearable Eye-Tracking Cost-Effective?

Why is a wearable eye tracker considered cost-effective? Likely, because the cost of acquiring one is quite low: between a few hundred euros for an open-source system and around €20k for a commercial system. However, this “cheap” acquisition cost is easily offset by the cost of manual labour needed for the eye-tracking data analysis. Unfortunately, most of the data analysis for wearable eye-tracking research is still done manually because there are few automatic analysis tools available (see e.g., Niehorster, Hessels, et al., 2020, for a discussion and examples). The time needed to analyse an eye-tracking recording of, say, 10 minutes, can easily exceed multiple hours depending on the specific question being asked (see e.g., Benjamins et al., 2018). In this regard, wearable eye-tracking can hardly be considered cost-effective.

## Is Wearable Eye-Tracking Easy to Apply?

It is true that for some wearable eye trackers, it is easy to put one on and start a measurement. But “easy to apply” suggests that the wearable eye tracker is easy to use for drawing conclusions on perception, cognition, and so forth. We think that this is not the case because (a) clear theoretical reasons for conducting wearable eye-tracking research are often lacking, (b) important concepts in eye-tracking research are often not or ill-defined, and (c) signal processing and analysing where someone looked in the world is problematic.

First, the theoretical reasons put forward for conducting eye-tracking research in the “real world” are often superficial. Many researchers motivate their “real-world research” by invoking greater “ecological validity,” yet fail to make clear what the relevant characteristics are of the behaviour of interest and why these could not be elicited in a laboratory (Holleman et al., 2020a). This results in an overestimation of what one may learn from a wearable eye-tracking study in the “real world.” What is often lacking is a theory or model about why the eye movements are relevant in the specific research setting. Consider, for example, a 1-hour recording of a teacher’s eye movements in the classroom. How does one interpret the eye-tracking data without an idea of whether eye movements (or gaze direction) are a bottleneck for the teacher’s behaviour, particularly in the absence of a benchmark?

Second, important concepts in eye-tracking research such as “fixations” and “saccades” are often not or ill-defined. This is crucial, as we have shown that even expert eye-movement researchers do not always agree about their definitions (Hessels et al., 2018). For example, one definition of “fixation” allows for the situation where an object is continuously looked at while it moves with respect to the world, while other definitions do not. Thus, a “fixation” in one study need not be comparable with a “fixation” in another study. This is particularly problematic when one study uses wearable eye trackers, while another uses world-fixed eye trackers. At best, this engenders confusion. At worst, this leads to nonsensical comparisons.

Third, there are data analysis problems. One example is automatic fixation classification. Wearable eye trackers report gaze direction with respect to the head, while world-fixed eye trackers report gaze position on, for example, a computer screen. If, in a world-fixed eye-tracking experiment, the visual stimuli are static, only fixations, saccades, and blinks occur. During fixations, an area of the screen is continuously foveated, while during saccades a new area of the screen is brought to the fovea. Fixations and saccades are easy to separate using, for example, a threshold on the gaze-velocity signal. In wearable eye-tracking research, however, objects may move with respect to the world, and participants can move with respect to the world. This results in a gaze-direction signal that may contain saccadic, smooth pursuit, fixational, and vestibulo-ocular reflex (VOR) components, among others. Separating these components is sometimes impossible from the gaze-direction signal alone, that is, distinguishing gaze on an object, while the participant moves from gaze on an object that moves with respect to the world. Crucially, automatic analysis tools to do this are not available yet in commercial software, meaning they are unavailable to a large proportion of wearable eye-tracking researchers.

Furthermore, the interpretation of the signal of a wearable eye tracker is difficult because it is a two-dimensional signal (gaze direction with respect to the head). Yet, many researchers require a three-dimensional signal (gaze location on an object in the world). Usually, researchers “solve” this problem by manually coding where a participant looked in the world from the gaze signal overlaid on the video recorded from the scene camera (which films part of the world in front of the participant). While manual coding may be useful, it is time-intensive and difficult to reproduce. Automatic tools to accomplish mapping the two-dimensional gaze direction to a three-dimensional gaze location in the world are still in their infancy. In a sparse world (e.g., a laboratory), the problem can be reduced by using the assumption that people usually look at objects. In more complex and larger environments (e.g., a classroom, mega store, or traffic), it is a difficult problem. It is exacerbated by the fact that the gaze direction error, if converted to a spatial deviation, scales with the distance to the object.

In sum, there are theoretical, conceptual, and methodological hurdles to overcome when conducting wearable eye-tracking research. We appreciate that some of these concerns can likewise be raised for many laboratory eye-tracking studies, for example, the problem of missing or ambiguous definitions and the lack of models that yield predictions at the level of the eye movements. However, we believe these problems are particularly evident in wearable eye-tracking research. Wearable eye trackers are appealing for nontechnical people, as many of the problems of building and fine-tuning laboratory eye-tracking setups and programming experiments can be circumvented. The wearable eye tracker can be applied right off the shelf in the “real world.” However, given the concerns outlined earlier, we conclude that wearable eye trackers may seem, but by no means are, “easy to apply.”

## Do Wearable Eye Trackers Reliably Measure Gaze Direction?

The last characteristic that we contest is that wearable eye trackers provide a reliable measure of gaze direction. In a recent study, we investigated the data quality (i.e., reliability, validity) of state-of-the-art wearable eye trackers (Niehorster, Santini, et al., 2020). Importantly, we studied data quality of these eye trackers in realistic conditions which one might readily encounter when conducting wearable eye-tracking research in the “real world.” One important problem is that the eye tracker may move slightly with respect to the head. Such movement may occur when one walks, talks, smiles, or when participants touch the eye-tracking glasses or their face. We show that most state-of-the-art eye trackers are not robust to movements of the eye tracker with respect to the head. This can result in large errors in the gaze-direction signals as well as tracking loss. Large errors in the reported gaze direction constrain the degree to which gaze to separate objects can be distinguished. Yet, we hardly find empirical values in the wearable eye-tracking literature for the observed errors in relation to the distance between objects of interest. Clearly, the reliability of wearable eye trackers in estimating gaze direction is overestimated.

## Impact

Wearable eye trackers have limitations. One that is often noted is that experimental design in wearable eye-tracking research in the “real world” is potentially more difficult than in a classical laboratory because one cannot control what happens in the “real world.” However, there are many other limitations we have highlighted here which often go unnoticed. Having taught in many international eye-tracking courses, we have seen the impact of overestimation of the ease of use, opportunities and the data quality of wearable eye trackers, and underestimation of the process of signal processing and data analysis. We often see PhD candidates and postdocs who have spent a number of years collecting data in the “real world,” only to find out that they have no realistic means of analysing the data or that their eye-tracking data does not allow them to answer their research question at all. The context is often similar: They are one of the first in their laboratory to work with such wearable technology and are often funded through an “innovative” grant application. Of course, such situations may also occur for PhD candidates and postdocs using “world-fixed” eye trackers. However, analysis of data from wearable eye trackers is much more complicated, and there are barely any (automatic) methods or established procedures for beginning researchers to rely on. The impact is therefore greater for wearable eye-tracking research. Without realistic expectations of what wearable eye trackers (and wearable technology in general) may deliver, research funds and the careers of young researchers are potentially wasted.

We see two potential solutions to the problem of overestimating wearable technology for understanding perception, cognition, and so forth, in the “real world.” The first is that claims about the potential of wearable technology should always be made in the context of convincing empirical examples. We realise that this is perhaps an idealistic solution. The second solution seems simple but requires investment from researchers. However, it is a sustainable solution. The solution is education on the use of wearable technology, for students, PhD candidates, postdocs, and professors alike. We encourage everyone who is interested in wearable eye tracking to dig deep into the technique first. Only then should it be applied to “real-world” scientific problems.
